# Non-traditional small companion mammals in Spain as reservoirs of antimicrobial-resistant *Staphylococci*

**DOI:** 10.3389/fvets.2024.1378346

**Published:** 2024-08-09

**Authors:** Ana Marco-Fuertes, Clara Marin, José Villora-Gonzalez, Concepción Gimeno-Cardona, Violeta Artal-Muñoz, Santiago Vega, Laura Montoro-Dasi

**Affiliations:** ^1^Facultad de Veterinaria, Instituto de Ciencias Biomédicas, Universidad Cardenal Herrera-CEU, CEU Universities, Valencia, Spain; ^2^Selvätica Veterinary Clinic, Valencia, Spain; ^3^Servicio de Microbiología, Consorcio Hospital General Universitario de Valencia, Valencia, Spain; ^4^Universidad de Valencia, Facultad de Medicina, Valencia, Spain

**Keywords:** antimicrobial resistance, methicillin resistance, non-traditional companion animals, small mammals, *Staphylococcus* spp.

## Abstract

**Introduction:**

The increasing prevalence of antimicrobial resistance (AMR) and multidrug resistance (MDR) in microorganisms poses a significant concern in both human and veterinary medicine. Non-traditional companion animals (NTCAs), particularly popular amongst households with children, play a crucial role in AMR epidemiology due to their rising population. Indeed, it is known that some of these animals may act as reservoirs of zoonotic pathogens and thus be able to spread and transmit them to family members, along with their AMR, through their shared environment. It is therefore imperative to address this concern with the involvement of human, animal and environmental health professionals. This pilot study aimed to assess the prevalence and AMR patterns of *Staphylococcus* spp. strains obtained from commensal mucosal and skin infection samples in NTC small mammals, with a focus on strains like methicillin-resistant *Staphylococcus* spp. (MRS) that are critical in public health.

**Methods:**

For this purpose, 81 animals of different small mammal species were sampled, assessing antimicrobial susceptibility to 27 relevant antimicrobial agents (AMAs) in human health using minimum inhibitory concentration assays, and interpreting them according to EUCAST and CLSI guidelines. The isolated *Staphylococc*i strains were identified by MALDI-TOF, with the predominant species being *Mammalicoccus sciuri* and *Staphylococcus aureus*.

**Results and discussion:**

Including all strains isolated, AMR was observed against all 27 AMAs, including six last-resort AMAs in human medicine. Additionally, over 85% of the strains exhibited MDR. These findings underscore the need to monitor AMR and MDR trends in companion animals and emphasise the potential role of NTCAs in spreading resistance to humans, other animals, and their shared environment, calling for a comprehensive “One Health” approach.

## 1 Introduction

Non-traditional companion animals (NTCAs), including small mammals (such as rabbits or ferrets), snakes, lizards or exotic birds, currently account for almost 30% of all companion animals in Europe. In particular, there has been a remarkable increase in the number of small mammals, to 29 million in European households today (1).

Small mammals, such as rabbits, guinea pigs or rodents, are considered ideal companion animals for children because of their manageable size, relatively easy maintenance and low risk of injury. They are socially interactive and can bond with children, providing opportunities to learn about responsibility and animal behaviour. These animals adapt easily to small spaces, making them easy to care for in domestic settings, and their presence offers children the chance to learn about nature and the basic needs of living things (2). For this reason, this growing trend in keeping NTCAs favours their close contact with their owners, becoming particularly important in households shared with at-risk populations (3). In addition, it has been observed that these animals can harbour different microorganisms, such as commensal and pathogenic bacteria, and transmit them together with their antimicrobial resistance (AMR) (4).

AMR is characterised by the ability of microorganisms to evolve over time and become resistant to the drugs used to fight the infections they induce (5, 6). This is especially critical due to the emergence of multidrug resistant (MDR) strains, which are strains of bacteria that have developed resistance to several classes of antimicrobial agents (AMA) (5), and therefore limited resources are currently available for effective intervention (7). In fact, due to the challenges posed by AMR and MDR in both human and veterinary medicine, the World Health Organisation (WHO) has declared them as one of the major threats to public health today (6), as these AMRs are not exclusive to a single species and can spread through the shared environment between humans and animals, underlining the need to address this issue through a “One Health” strategy (4, 8).

Traditionally, the importance of AMR in livestock has been studied, together with its association with farmers (9), but few studies have been conducted in domestic animals despite the importance of its impact on owners, who are often children. In fact, different AMR monitoring and surveillance programmes have been implemented in the European Union (EU) for zoonotic and commensal bacteria in food-producing animals by the European Food and Safety Authority (EFSA) (10), and in human medicine by the European Centre for Disease Prevention and Control (ECDC) (11). However, particularly in the EU, each Member State has additionally implemented its own programmes, for example the National Antimicrobial Resistances Plan (PRAN, from its Spanish acronym Plan Nacional Resistencia Antibióticos) in Spain (12). Currently, there is a need to homogenise all these programmes to compare the available data and establish the current AMR epidemiological situation, including in food-producing and companion animals. For this reason, the EU intends to set up the European Antimicrobial Resistance Surveillance Network in Veterinary medicine (EARS-Vet) (13, 14), but only traditional companion animals, such as dogs and cats, are included in this project, leaving aside NTCAs.

In AMR epidemiological studies, the species within the *Staphylococceae* family are of special relevance as they are part of the commensal microbiota of the skin and mucosa of animals and humans, yet they are also considered opportunistic pathogens that can cause both human and animal infections (15). This family is divided into coagulase-positive *Staphylococci* (CoPS) and coagulase-negative *Staphylococci* (CoNS). Both groups have been identified as pathogenic bacteria with significant potential to cause severe infections in both human and veterinary medicine (16). In particular, one of the main bacteria monitored worldwide is *Staphylococcus aureus* (CoPS), which is one of the most widely distributed *Staphylococcus* species, as it is widely present in both humans and animals and has also been designated by the WHO as one of the high priority bacteria for research and development of new AMAs due to its high resistance (17). This is of special importance due to the emergence of methicillin-resistant *Staphylococcus aureus* (MRSA) strains, one of the high priority pathogens listed by the WHO (17). However, the need to monitor all methicillin-resistant *Staphylococci* (MRS) strains due to its public health importance must be highlighted (18). In addition, it is important not to forget CoNS strains, as many of them have also been reported to be methicillin-resistant, potentially leading to therapeutic failures in the treatment of infections caused by these challenging strains (19, 20).

Nevertheless, despite all this information, there are no available programmes focused on NTCAs, although they are considered carriers of *Staphylococcus* spp. and can transmit them to their owners. Furthermore, few studies on NTCAs have been carried out in Europe, and the few that have been done have focused mainly on rabbits or rodents, the most popular NTC small mammals (21–24). Therefore, more studies are needed to achieve a global vision of AMR and MDR in the different animal species included in this bacterial group. Thus, to obtain a comprehensive initial overview, the aim of this pilot study was to assess the prevalence and AMR patterns of *Staphylococcus* spp. strains isolated from commensal mucosal samples and skin infection samples taken from NTC small mammals. Additionally, the study also aimed to investigate the presence of MDR and MRS in these strains.

## 2 Materials and methods

### 2.1 Experimental design

The Animal Ethics Committee of the UCH-CEU University (research number CEEA 22/04) reviewed and approved the present animal study carried out in Valencia Region.

For this purpose, an important veterinary centre (VC), which exclusively deals with exotics and NTCAs, was invited to participate on a voluntary basis. This centre deals with almost 70% of the exotic animal population of the Valencian Community, as it receives animals derived from several clinics and hospitals in Valencia, which makes it an exhaustive and representative sampling site for the study.

### 2.2 Epidemiological data collection

With the aim of taking the samples and collecting all epidemiological information on these sampled animals, informed consent was first requested from all animal owners. First, an epidemiological questionnaire was filled out by the veterinarians in the practise, which contained details on the origin of the animals. The second part provided general data on the animals including their sex, age, whether they shared the household with other animals and whether they had outdoor access. Lastly, the third and final section of the questionnaire focused on clinical data related to the animals. It included information on whether the animal had any chronic diseases, whether it was currently taking any daily medication, details about its most recent AMAs treatment, and a record of specific AMAs administered throughout its lifetime. In addition, to study the impact that AMAs have in the development of AMR and MDR, four groups were made to classify animals depending on when they were last treated: (I) Never; (II) In the last 6 months: (III) In the last month; (IV) Under treatment at the time of sampling. The questionnaire is available in the Supplementary material.

### 2.3 Sample collection

To study the prevalence of *Staphylococcus* spp., its AMR patterns and multidrug resistance from NTC small mammals, samples were collected between January and June 2023, from any animals attending the VC. Two types of samples were taken: for the first, a swab (Cary-Blair sterile transport swabs, DELTALAB, Barcelona, Spain) was introduced in the nasal and then in the auricular cavity, from healthy asymptomatic small mammals, based on previous studies (25–27). To verify the health status of the animals, the veterinarians carried out a clinical examination, assessing vital signs, such as corporal temperature (Tª), and cardiac, respiratory and corporal condition (28), to ensure that they were within normal ranges, so that they could be classified as asymptomatic healthy animals. The second sample was taken to isolate infection-causing *Staphylococcus* spp. To this end, a swab (Cary-Blair sterile transport swabs, DELTALAB, Barcelona, Spain) was taken from animals with active skin infections, which was introduced in apparently skin infected wounds.

For further analyses, all samples were transported to the microbiology laboratory at the Faculty of Veterinary Sciences of the University CEU Cardenal Herrera, preserved in Cary-Blair transport medium and refrigerated at ≤4°C within 24 h of collection.

### 2.4 *Staphylococcus* spp. isolation and identification

The sample swabs were subjected to pre-enrichment in buffered peptone water (BPW; Scharlau, Barcelona, Spain) at a ratio of 1:10 vol/vol and then incubated at 37 ± 1°C for 24 h. Then, the suspension was seeded on non-specific agar, Columbia CNA agar with 5% Sheep Blood, Improved II (BD, Becton Dickinson, Madrid, Spain), and incubated at 37 ± 1°C for 24 to 48 h. Observation of the plates occurred at both the 24 and 48 h marks. Suspected colonies showing typical *Staphylococcus* spp. morphology on blood agar, along with a positive catalase test result, were identified by MALDI-TOF MS Biotyper System (Bruker Daltonics, Madrid, Spain) at the Microbiology Service of the *Consorcio Hospital General Universitario de Valencia*. The Standard Bruker criteria, ranging from 0.00 to 3.00, were used to interpret the results obtained (29). These scores are classified into three groups: the range of 2.00–3.00 means a high confidence identification by species; ranges between 1.70 and < 2.00 provided a low confidence identification by species (only reliable to genus level); and finally, ranges <1.70 do not provide a reliable identification. Only scores above 2.00 were included in this study.

### 2.5 Antimicrobial susceptibility testing

The antimicrobial susceptibility testing, which included important AMAs for public health, was performed following the protocol described in previous studies (30). In addition, MDR was defined as acquired resistance to at least one agent in three or more antimicrobial classes (5).

However, since little is known on the epidemiological status of NTC small mammals regarding their AMR for *Staphylococcus* spp., and there is no specific monitoring and surveillance programme for their AMR, two panels of AMAs were performed. The first panel, carried out with the GPALL1F Gram-Positive Sensititre Plate (Thermo Scientific™ Sensititre™, Madrid, Spain) (Table 1), included 20 AMAs of public health relevance and clinically important AMAs for human medicine and included in the EARS-Vet programme (32). Additionally, the plate had two D-test wells, combining clindamycin (CLI) and erythromycin (ERY). These wells indicated whether the strain tested had inducible resistance to CLI in the presence of ERY, which could lead to therapeutic failure. Interpretation of the results was performed following the guidelines of the Spanish Society of Infectious Diseases and Clinical Microbiology (SEIMC) (33). The second panel, which was performed with the EU Surveillance *Staphylococcus* EUST2 Sensititre Plate (Thermo Scientific™ Sensititre™, Madrid, Spain) (Table 1), included the AMAs with relevance in public health set out in Decision (EU) 2023/1017 as regards the monitoring of MRSA in fattening pigs (34), the only available legislation currently regarding this bacterium in the EU.

**Table 1:** Antimicrobial agents, latest WHO antimicrobial classification and their studied concentrations included in GPALL1F Gram-Positive Sensititre Plate and EU Surveillance *Staphylococcus* EUST2 Sensititre Plate (both Thermo Scientific™ Sensititre™, Madrid, Spain).

Antimicrobial agent group	Antimicrobial agent	Abbreviation	WHO	Concentration
Aminoglycosides	Gentamycin^1^ Kanamycin^1,2^ Streptomycin^1,2^	GEN KAN STR	CIA CIA CIA	2-16 μg/mL 4-32 μg/mL 4-32 μg/mL
Amphenicols	Chloramphenicol^1,2^	CHL	HIA	2-16 μg/mL
Ansamycins	Rifampicin^1,2^	RIF	CIA	0.015-4 μg/mL
Cephalosporins	Cefoxitin^1,2^	CXI	HIA	0.5-16 μg/mL
Folate Inhibitor Pathway	Trimethoprim / Sulfamethoxazole^1^	TRS	HIA	1/19-8/152 μg/mL
	Trimethoprim^2^ Sulfamethoxazole^2^	TMP SXM	HIA HIA	1-16 μg/mL 64-512 μg/mL
Fusidates	Fusidic acid^2^	FUS	HIA	0.25-4 μg/mL
Glycopeptides	Vancomycin^1,2^	VAN	NA	0.25-32 μg/mL
Glycylcyclines	Tigecycline^1^	TIG	NA	0.03-0.5 μg/mL
Lincosamides	Clindamycin^1,2^	CLI	HIA	0.12-4 μg/mL
Lipopeptides	Daptomycin^1^	DAP	NA	0.5-4 μg/mL
Macrolides	Erythromycin^1,2^	ERY	CIA	0.25-8 μg/mL
Nitrofurans	Nitrofurantoin^1^	NIT	NA	32-64 μg/mL
Oxazolidinones	Linezolid^1,2^	LIN	NA	1-8 μg/mL
Penicillins	Ampicillin^1^	AMP	HIA	0.25-8 μg/mL
	Oxacillin + 2 % NaCl^1^	OXA+	HIA	0.25-4 μg/mL
	Penicillin^1,2^	PEN	HIA	0.06-8 μg/mL
Pleuromutilins	Tiamulin^2^	TIA	IA	0.5-4 μg/mL
Pseudomonic acid	Mupirocin^2^	MUP	NA	0.5-256 μg/mL
Quinolones	Levofloxacin (FQ) ^1^	LEV	HPCIA	0,25-4 μg/mL
	Ciprofloxacin (FQ) ^1,2^	CIP	HPCIA	1-2 μg/mL
	Moxifloxacin (FQ) ^1^	MOX	HPCIA	0.25-4 μg/mL
Tetracyclines	Tetracycline^1,2^	TET	HIA	0.05-16 μg/mL
Streptogramins	Quinupristin / Dalfopristin^1^	QUD	HIA	0.5-4 μg/mL
D-test	Erythromycin (E) + Clindamycin (C)	DT^1^		4 μg/mL (E) + 0.5 μg/mL (C)

FQ: Fluoroquinolone. WHO: World Health Organisation [This column indicates the last updated of the classification of medically important antimicrobials authorised by WHO for human and animal use in order to protect public health, updated in 2023 (31)]. HIA: highly important antimicrobial. CIA: critically important antimicrobial. HPCIA: highest priority critical important antimicrobial. NA: not authorised for animal use (31). ^1^antimicrobial agents included in GPALL1F Gram-Positive Sensititre Plate. ^2^antimicrobial agents included in EU Surveillance *Staphylococcus* EUST2 Sensititre Plate. Additionally, both plates had two positive control wells.

To this end, analyses were performed according to the manufacturer’s instructions (ThermoFisher Scientific™, Madrid, Spain) (35). Manual reading of the plates was performed using a Sensititre Vizion (Thermo Scientific™ Sensititre™ Vizion™ Digital MIC Viewing System, ThermoFisher Scientific, Madrid, Spain).

All the results were interpreted based on the guidelines from the European Committee on Antimicrobial Susceptibility Testing (EUCAST) in its latest report (14th ed., 2024) (36). MRS strains were examined by assessing AMR against cefoxitin (the antibiotic used for screening MRSA and methicillin-resistant coagulase-negative *Staphylococci* (MR-CoNS) strains), and agains oxacillin + 2% NaCl, the antibiotic used for screening methicillin-resistant *Staphylococcus pseudintermedius* (MRSP). However, as some MIC values of these antibiotics for screening MR-CoNS and MRSP are not currently available in EUCAST, the Clinical and Laboratory Standards Institute (CLSI) recommendations, specified in M100 (37) and VET01 (38), were followed in those cases.

### 2.6 Statistical analysis

Once the analyses were complete and all study data had been obtained, they were analysed using a generalised linear model (GLM) with a probit link function, assuming a binomial distribution. This was done to examine the influence of intrinsic and external epidemiological factors of each animal on the occurrence of AMR and MDR patterns in small mammalian *Staphylococcus* spp. The objective of this analysis was to determine associations with categorical variables, including animal origin, sex, cohabitation with other animals, relationship with animals outside the household, and clinical information regarding chronic diseases, daily medication, and previous antibiotic treatments. A significance level of *p*-value ≤0.05 was considered indicative of a statistically significant difference. Statistical analyses were performed using the R software (version 4.3.1) packages EMMs (39), car (40) and multicompView (41).

## 3 Results

### 3.1 Epidemiological results

In the present study, 81 small mammals of nine different species were sampled. All of them and the number of samples taken by each animal species are in Table 2.

**Table 2:** Number and percentage of the different animal species sampled.

Animal species (common name)	*n* (%) of animals sampled
*Oryctolagus cuniculus* (European rabbit)	51 (63.1)
*Cavia porcellus* (Guinea pig)	18 (22.2)
*Rattus norvegicus* (common rat)	3 (3.7)
*Cricetinae* (common hamster)	3 (3.7)
*Gerbillinae* (gerbil)	2 (2.5)
*Chinchilla laniguera* (chinchilla)	1 (1.2)
*Erinaceinae* (hedgehog)	1 (1.2)
*Mustela putorius furo* (ferret)	1 (1.2)
*Petaurus breviceps* (sugar glider)	1 (1.2)
Total	81

n: number, %: percentage.

**Figure 1:**
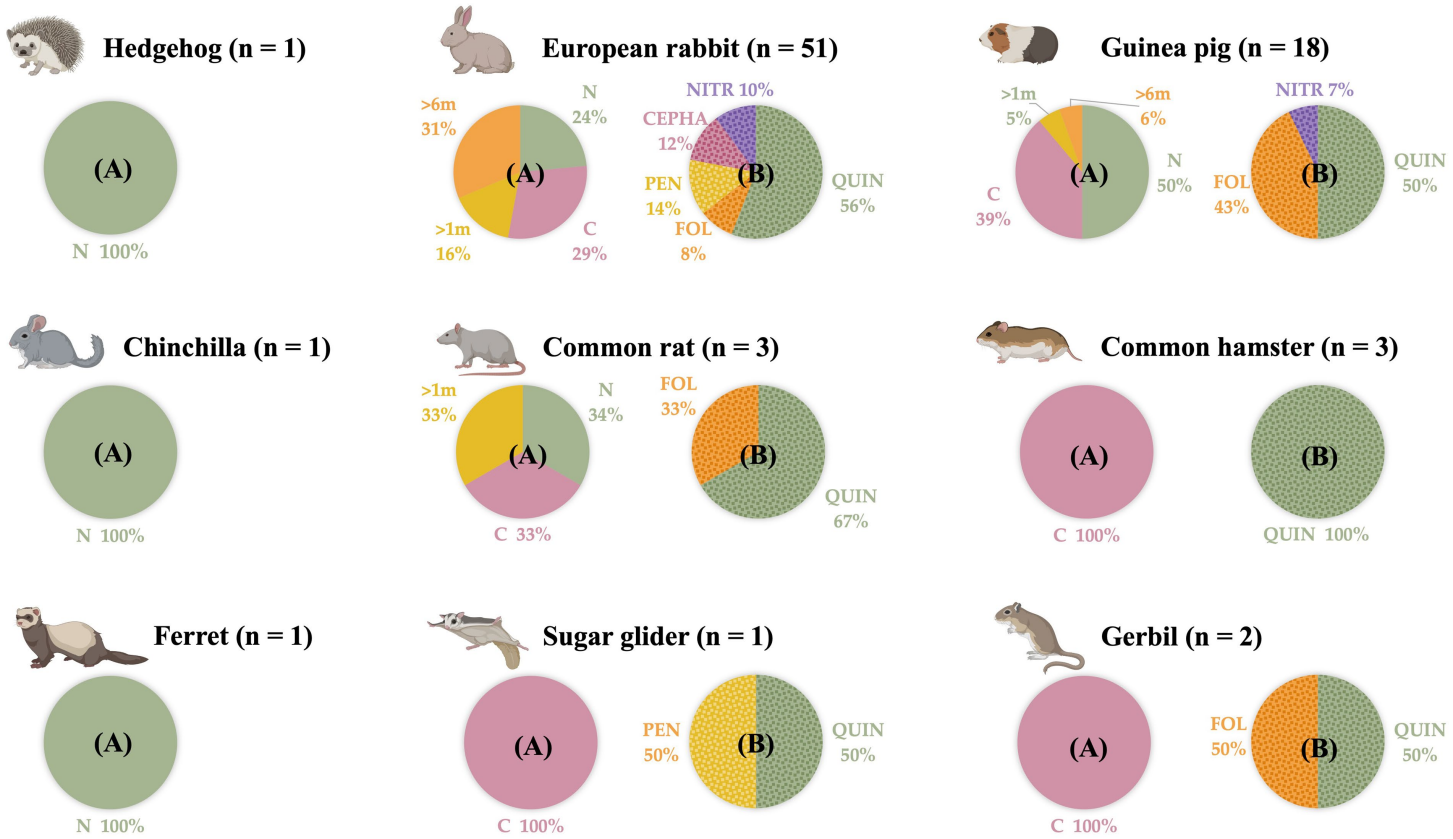
Distribution by animal species of the small mammal population studied, according to when they were last treated with antimicrobial agents and with which antimicrobial agents group. n: number of animals sampled. **(A)** Moment of the last antimicrobial agents administration. N: never. C: currently. >1 m: in the last month. >6 m: in the last 6 months. **(B)** Antimicrobial agents groups administered in the study population at some point of their lives. QUIN: quinolones. FOL: folate inhibitor pathway. CEPHA: cephalosporins. PEN: penicillins. NITR: Nitroimidazoles. (Created by Biorender).

First, epidemiological information, including gender and age, was gathered on all the animals in this study. Nevertheless, due to the diverse nature of the study population, which includes several animal species from different families, the data are not directly comparable. Regarding their style-life, 61.7% (50/81) of the animals cohabited in the same household with other animals, but none of them went out of their house. Secondly, according to the clinical information gathered, 68% (55/81) of the animals presented a chronic disease, and 14.8% (12/81) were taking daily medication. Finally, of all the animals, 70.4% (57/81) had been previously treated with AMAs at some point in their lives. The data presented in Figure 1 show the AMAs treatment history of the study population, detailing the specific AMAs group and the date of the last treatment.

### 3.2 *Staphylococcus* spp. prevalence

Of the 81 specimens sampled, 72 were asymptomatic animals and 9 presented a skin infection. Of all of them, the total prevalence of *Staphylococcus* spp. was 48.2% (39/81), of which 42% (34/81) and 6.2% (5/81) were commensal and infection-causing *Staphylococcus* spp., respectively. All *Staphylococcus* spp. isolated from each of the small mammals, together with the type of sample from which they were derived, are listed in Table 3.

**Table 3:** Prevalence of *Staphylococcus* species isolated from commensal mucosa and skin infection samples from small mammals.

Type of sample	Prevalence of *S.* by class	*S.* species	*N* and (%) prevalence of each *S*. species	(*N*) of *S.* strains per animals’ species
Commensal mucosa	CoPS – 17.6%	*S. aureus*	5 (14.6)	*Oryctolagus cuniculus* (3) *Gerbillae* (1) *Rattus norvegicus* (1)
		*S. pseudintermedius*	1 (3)	*Oryctolagus cuniculus* (1)
	CoNS – 82.4%	*S. borealis*	2 (5.9)	*Cavia porcellus* (2)
		*S. cohnii*	3 (8.8)	*Oryctolagus cuniculus* (3)
		*S. epidermidis*	1 (3)	*Oryctolagus cuniculus* (1)
		*S. haemolyticus*	3 (8.8)	*Oryctolagus cuniculus* (2) *Cavia porcellus* (1)
		*S. hominis*	1 (3)	*Mesocricetus auratus* (1)
		*S. microti*	3 (8.8)	*Cavia porcellus* (2) *Ernaceinae* (1)
		*S. saprophyticus*	2 (5.9)	*Oryctolagus cuniculus* (1) *Mesocricetus auratus* (1)
		*S. sciuri* ^1^	6 (17.7)	*Cavia porcellus* (4) *Mesocricetus auratus* (1) *Oryctolagus cuniculus* (1)
		*S. warneri*	2 (5.9)	*Oryctolagus cuniculus* (2)
		*S. xylosus*	5 (14.6)	*Cavia porcellus* (1) *Oryctolagus cuniculus* (4)
Skin infection	CoPS – 60%	*S. aureus*	3 (60)	*Oryctolagus cuniculus* (1) *Cavia porcellus* (1) *Gerbillae* (1)
	CoNS – 40%	*S. epidermidis*	1 (20)	*Oryctolagus cuniculus* (1)
		*S. xylosus*	1 (20)	*Oryctolagus cuniculus* (1)

N: number of strains isolated. CoPS: coagulase-positive *Staphylococcus*. CoNS: coagulase-negative *Staphylococcus. S.: Staphylococcus*. ^1^Due to a new phylogenomic study on the family *Staphylococcaceae, S. sciuri* now belongs to a new genus and has been renamed as *Mammaliicoccus sciuri*. However, in biochemical and MALDI-TOF identification, it is still identified as *S. sciuri*.

### 3.3 Antimicrobial susceptibility in *Staphylococcus* spp. strains

#### 3.3.1 Methicillin-resistant strains

In the present study, all MRS strains came from the commensal bacteria isolates, and none of the strains isolated from active skin infections showed methicillin resistance. MRS strains represented a 14.7% (5/34), belonging each one to a different species: *Mammaliicoccus sciuri, S. aureus, S. xylosus, S. haemolyticus* and *S. epidermidis*.

#### 3.3.2 Antimicrobial resistance profile

**Figure 2:**
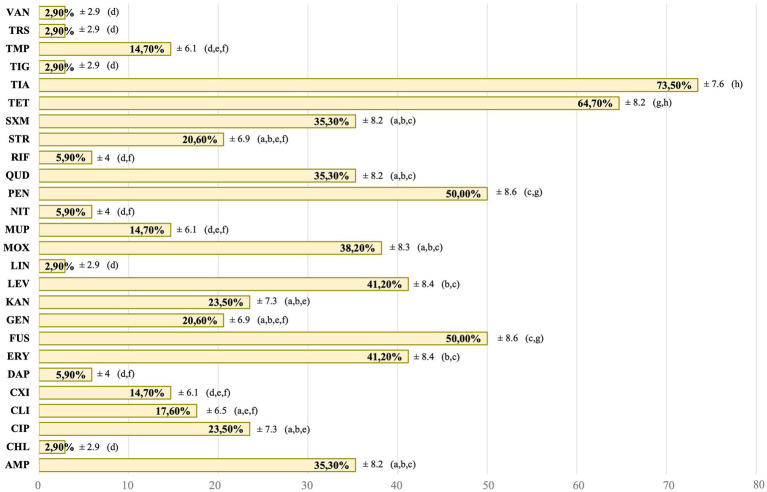
Antimicrobial resistance of the total commensal *Staphylococcus* spp. strains. AMP: ampicillin. CHL: chloramphenicol. CIP: ciprofloxacin. CLI: clindamycin. CXI: cefoxitin. DAP: daptomycin. ERY: erythromycin. FUS: fusidic acid. GEN: gentamycin. KAN: kanamycin. LEV: levofloxacin. LIN: linezolid. MOX: moxifloxacin. MUP: mupirocin. NIT: nitrofurantoin. PEN: penicillin. QUD: quinupristin/dalfopristin. RIF: rifampicin. STR: streptomycin. SXM: sulfamethoxazole. TET: tetracycline. TIA: tiamulin. TIG: tigecycline. TMP: trimethoprim. TRS: trimethoprim/sulfamethoxazole. VAN: vancomycin. a–h: different letters indicate significant statistically differences between the antimicrobial agents studied.

Regarding the *Staphylococcus* spp. and *Mammaliicoccus* spp. strains isolated from commensal samples in the present study, all the strains (34/34) showed AMR to at least one of the 27 AMAs studied, and 85.3% (29/34) were MDR. Of all the commensal strains, 17.6% (6/34) were positive to the D-test performed. The AMR values for the AMAs groups, where more than one AMAs was studied, were 34.3% for quinolones, 29.4% for penicillins, 17.6% for folate inhibitor pathway. For the remaining AMAs groups, only one AMA from each group was studied, so Figure 2 shows the AMR for each AMA individually, with the exception of oxacillin, which was tested against a single strain of *S. pseudintermedius* and found to be susceptible. The AMR observed for each of the isolates, these are detailed in the Supplementary Table 1.

**Figure 3:**
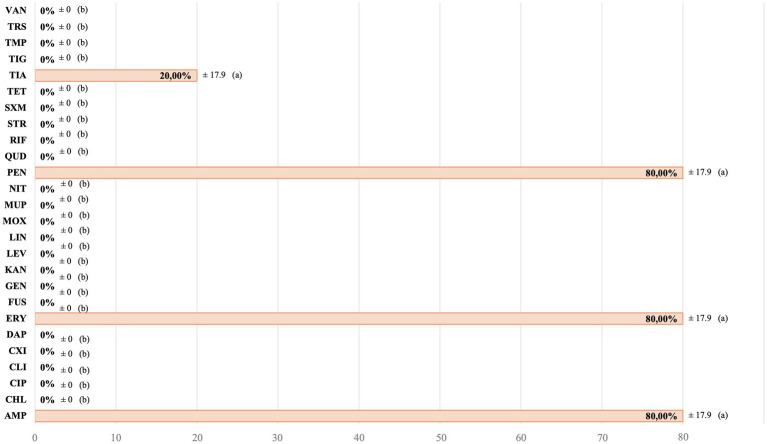
Antimicrobial resistance of the total infection-causing *Staphylococcus* spp. strains. AMP: ampicillin. CHL: chloramphenicol. CIP: ciprofloxacin. CLI: clindamycin. CXI: cefoxitin. DAP: daptomycin. ERY: erythromycin. FUS: fusidic acid. GEN: gentamycin. KAN: kanamycin. LEV: levofloxacin. LIN: linezolid. MOX: moxifloxacin. MUP: mupirocin. NIT: nitrofurantoin. PEN: penicillin. QUD: quinupristin/dalfopristin. RIF: rifampicin. STR: streptomycin. SXM: sulfamethoxazole. TET: tetracycline. TIA: tiamulin. TIG: tigecycline. TMP: trimethoprim. TRS: trimethoprim/sulfamethoxazole. VAN: vancomycin. a,b: different letters indicate significant statistically differences between the antimicrobial agents studied.

For all infection-causing *Staphylococcus* spp. isolated from animals with active skin infections, all of them (5/5) were resistant to at least one of the studied AMAs, and 40% (2/5) were MDR. Moreover, the D-test performed in these strains was positive in 80% (4/5) of them. For AMAs groups with more than one AMA studied, AMR rates were 60% for penicillins and 20% for quinolones. For folate inhibitor pathway, no AMRs were shown. Figure 3 shows the AMR for each individual AMA studied. Regarding the AMR observed in each of the isolates, these are detailed in the Supplementary Table 1.

Furthermore, no relationship was observed between the epidemiological and clinical data collected in the questionnaire, and the occurrence of AMR and MDR, neither in commensal nor in those infection-causing *Staphylococcus* spp. strains (*p*-value >0.05).

Lastly, no discernible pattern in overall AMR trends was observed in this study. Amongst the 39 *Staphylococcus* spp. and *Mammaliicoccus* spp. isolates, 37 distinct AMR patterns were identified, indicating a diverse range of AMR profiles. Only two patterns were duplicated, one to folate inhibitor pathways together with pleuromutilins and quinolones, and the second to fusidates, pleuromutilins and tetracyclines, both in two commensal *Staphylococcus* spp. isolates (2/39). The list of AMR patterns can be found in the Supplementary Table 1.

## 4 Discussion

The *Staphylococcaceae* family is one of the most common bacteria overall, and particularly amongst gram-positive bacteria, as this microorganism is part of the normal microbiota on the skin and mucous membranes of humans and most animals. Recently, new phylogenomic studies of this family have been carried out, relocating some *Staphylococcus* spp. into other genera, such as the former *S. sciuri*, now called *Mammaliicoccus sciuri*. However, the importance of this strain remains the same, as it is considered the evolutionary reservoir of the *mecA* gene, which encodes methicillin resistance. Encompassing all the bacterial species found in this study, the observed prevalence is consistent with that found in other studies of both NTC and free-living small mammals (21, 42, 43), with most of the isolates being CoNS. In the present study, *M. sciuri* was the most prevalent bacterium from commensal samples, followed by *S. aureus*. In skin infection isolates, *S. aureus* was the most prevalent species. Although a high variability was found, as 10 additional bacterial species have been observed, as reported in other studies carried out in small mammals (44). One of the hypotheses for this high diversity of *Staphylococci* in NTC small mammals could be their household environment. They share it with humans of all ages, and more than 60% of the animals shared it with other companion animals, of the same or other species such as dogs, that go outside daily, making the environmental microbiome in homes richer and thus favouring different bacterial species colonising the mucosa of NTCAs. Another possible reason could be the high rate (more than 70%) of previous antimicrobial treatment in the study population, which puts pressure on bacterial communities and may favour the growth of selected bacterial species.

Moreover, the rise of AMR and MDR in *Staphylococcus* spp. strains in veterinary medicine poses a global public health challenge. Research indicates that these resistant strains can persist in the environment and be transferred between animals and humans (45, 46). This underlines the need to assess the prevalence of such resistances in both commensal and pathogenic bacteria, requiring a comprehensive “One Health” approach. Addressing this problem is vital not only to avoid therapeutic failures in veterinary medicine, but also to safeguard human health, especially with the results observed in this study, where all the strains were resistant to at least one of the AMAs studied, and more than 85% of them were MDR, with a diverse range of AMR profiles, not following any discernible pattern. Similar results have been observed in other NTCAs (21, 47) and traditional companion animals (48), highlighting this global problem. In addition to MDR, the surveillance of MRS strains is crucial, mainly due to their resistance to common AMAs, which complicates the selection of effective treatments. Moreover, MRS strains are known for their ability to spread rapidly in health care facilities. In this study, MRS strains (14.7%) have been observed in both CoPS and CoNS, as reported in studies carried out in other countries, such as Austria (47) or Turkey (49), in dog, cats and NTCAs, which underlines the global need to monitor these strains.

Regarding each AMA, AMR observed against tiamulin (TIA) stands out above the others with 73.5%. TIA is an AMA exclusively used in veterinary medicine, particularly for food-producing animals, especially pigs and poultry, for which similar AMR rates have been observed (50). However, it is also approved for use, although to a lesser extent, in meat-producing rabbits (51), which may contribute to their use in rabbits kept as companion animals and not for production purposes. The following AMAs with higher AMR were tetracycline (TET; 64.7%) and fusidic acid (FUS; 50%), both AMAs belonging to the highly important antimicrobials (HIA) category in the latest WHO categorisation (31). It is therefore to be expected that higher percentages of AMR will be observed against these AMAs (52) and not against those belonging to higher categories. Other AMAs in this category, which are one of the first line treatments, are folate inhibitor pathways, such as trimethoprim (TMP), sulfamethoxazole (SMX) or the combination of both (trimethoprim-sulfamethoxazole, TRS). Although these AMAs can be administered separately, higher AMR resistance rates were seen individually (TMP, 14.7%; SMX, 35.5%) than those observed to TRS (2.9%) in combination, which highlights the importance of using this combination in veterinary medicine, until this therapeutic option is exhausted (53). However, higher AMR has been seen in this combination in traditional companion animals, reaching almost 50% (30, 54).

Of the AMAs studied, erythromycin (ERY) represents one of the first treatments of choice for *Staphylococcal* infections, especially in patients with penicillin allergies (55). The high AMR rates found in this study for ERY aligns with those found in other studies in small mammals (42), dogs and cats in Spain (48) and Canada (56), or in dogs and their owners in Italy (27), which indicates that first therapeutic options to treat these infections may begin to fail. For this reason, it is important to explore the AMR to other therapeutic options, such as clindamycin (CLI), a HIA category AMA, but used to treat community-acquired skin infections probably due to MRS (55). However, to evaluate whether this AMA can be used in the practise or not, the D-test should be performed, to confirm whether an inducible CLI resistance phenotype is present or not (57). In the present study, 17.6 and 80% of commensal and infection-causing strains, respectively, were positive to the D-test. This result indicates that, although CLI alone may appear to be effective, the bacteria can develop resistance during treatment, which can have serious implications for infection management, as AMR can compromise treatment (58). Moreover, inducible resistance to CLI confirms the macrolides, lincosamides, streptogramin ß and pleuromutilin (MLS_B-P) group resistance phenotype, as resistance genes which induce resistance to CLI can also induce resistance to MLS_B-P, which are antibiotics commonly used for the treatment of MRSA (59). This may be one of the reasons why high rates of AMR to these AMAs were observed in this study.

When all other therapeutic options fail, the last AMAs that can be used in veterinary medicine are the highest priority critical important antimicrobials (HPCIAs), including the quinolones (31), although in the study population, quinolones were the most administered AMAs group. In this study, the three quinolones evaluated: levofloxacin (LEV), ciprofloxacin (CIP) and moxifloxacin (MOX), are AMAs only approved for use in human but not in veterinary medicine in the EU (31). Therefore, the high AMR observed (34.3%) to this group, similar to that observed in another study in rabbits (21), is of concern due to the therapeutic failures it could pose, and the possibility of transmission of these AMRs to other pathogenic bacteria (60).

Finally, the last category available when all the others have failed is reserved for human medicine, and is not authorised for veterinary medicine, being more commonly known as last-resort AMAs (31). The AMAs of this category studied were vancomycin (VAN), tigecycline (TIG), linezolid (LIN), daptomycin (DAP), nitrofurantoin (NIT) and mupirocin (MUP). These AMAs are usually reserved for severe or life-threatening infections that do not respond to standard AMA therapies using the above categories. Regarding the AMR observed, a low prevalence of almost all AMAs was found, aligning with other studies conducted in small mammals in the Czech Republic (43) and in dogs, cats, and rabbits in Lithuania (61), but not for MUP. This AMA is for topical use only, utilised for complicated skin infections, including those caused by MRS, and for decolonising nasal carriers of *S. aureus*. Although given the importance of this AMA, a lower percentage of AMR should be observed, the prevalence reported in this study (14.7%) is within normal ranges, considering that in Spain the AMR for this AMA in CoNS isolates is around 40% and for *S. aureus* between 8 and 10%, in human medicine (62).

The present study is focused on assessing the prevalence and AMR patterns of *Staphylococcaceae* strains isolated from mucosal samples and skin infections in small mammals. The results highlight the high prevalence of AMR and MDR in small mammals, underlining the need for a comprehensive “One Health” approach to address this issue, as these animals share the domestic environment with humans and other animals. Moreover, the diversity of bacterial species and the high rate of previous antimicrobial treatments suggest significant selective pressure, which may favour the emergence of AMR. This research is an initial step for future initiatives to control and prevent the proliferation of AMR and MDR in NTCAs. However, further research is essential to validate our results in a larger and more representative study population.

## Data availability statement

The original contributions presented in the study are included in the article/Supplementary material, further inquiries can be directed to the corresponding authors.

## Ethics statement

The animal studies were approved by Animal Ethics Committees of the Universidad Cardenal Herrera-Ceu. The studies were conducted in accordance with the local legislation and institutional requirements. Written informed consent was obtained from the owners for the participation of their animals in this study.

## Author contributions

AM-F: Data curation, Formal analysis, Investigation, Methodology, Resources, Software, Visualization, Writing – original draft, Writing – review & editing. CM: Conceptualization, Funding acquisition, Investigation, Methodology, Project administration, Resources, Supervision, Validation, Visualization, Writing – review & editing. JV-G: Methodology, Resources, Supervision, Validation, Visualization, Writing – review & editing. CG-C: Investigation, Methodology, Resources, Supervision, Validation, Writing – review & editing. VA-M: Investigation, Methodology, Supervision, Validation, Writing – review & editing. SV: Conceptualization, Funding acquisition, Investigation, Project administration, Resources, Supervision, Validation, Visualization, Writing – review & editing. LM-D: Conceptualization, Formal analysis, Investigation, Methodology, Project administration, Resources, Supervision, Validation, Visualization, Writing – original draft, Writing – review & editing.
